# Identifying triggers of migraine in children and adolescents: Opportunities for intervention

**DOI:** 10.1111/dmcn.70394

**Published:** 2026-07-08

**Authors:** Kenneth J. Mack

**Affiliations:** ^1^ University of Wisconsin‐Madison Madison WI USA

## Abstract

**This commentary is on the original article by Zahran and Ahmed on pages** 1476–1481 **of this issue.**

For patients with migraine, health care providers usually focus on three aspects of treatment.^1^ We discuss pain control at the time of the acute attack. For patients with frequent headaches, we offer choices in preventive therapy. But perhaps the most important intervention we can offer is to discuss lifestyle and environmental factors that promote migraine, that is, triggers.

In the article by Zahran and Ahmed,^2^ the authors identified triggers in nearly 40% of their patients. An important part of this work is that it identifies an opportunity for us to intervene in patients' headache management. Educating patients about their potential triggers and risk factors may be the single most important item we offer in an initial office visit.

The most common trigger identified was stress, reported by 25% of patients. Stress may be a hard concept for many children to understand. If one substitutes the term ‘busy schedules’ then that concept can become a lot more relatable to some patients. This can open up specific discussions on how to deal with stress or busy schedules, such as adjusting work‐life balance; adequate time for relaxation and exercise; or finding a friend, parent, or a therapist to talk with about the stresses of the day.

Surprisingly, only about 3% of patients reported that insufficient sleep worsened their headaches, although other studies have identified sleep deprivation as a major trigger. One useful question to ask is, ‘How do you feel when you wake up in the morning?’ If patients say they feel tired, it provides a natural opening to discuss both the quality and quantity of their sleep. This is especially relevant for adolescents, who often prefer later bedtimes but must wake early for school.

As the authors note, school is a complex issue, and there may be many specific factors that are involved if we see a higher frequency of headaches during the school year than during vacation. Certainly, the relative decreased amount of sleep and busier schedules during the school year are factors. But in addition to that, loud noise and bright lights can also be contributing factors. And many patients with frequent headaches may have a social anxiety about going to school.

Approximately 8% of their patients reported a food or beverage trigger. Although one can provide a list of common triggers for people with migraines, sometimes individuals will have very specific and unexpected food triggers that are not very common. In my own experience, I have not found that food diaries or exclusion diets were very helpful, perhaps because of the complexity of our diets. Within the field of headache medicine, it is mildly controversial as to whether or not foods are specific triggers, or if instead, food cravings are part of the premonitory phase of the migraine attack.

In this study, the role of hormonal factors were not commonly identified. Many young females will notice an increase in their headache frequency as they go into puberty. And post‐pubertal females will often notice an increase in migraine headaches as their estrogen levels drop just prior to menses.

So even if the exact percentages of migraine triggers differ slightly between studies, the importance of this study is to recognize that triggers exist, and this is an actionable opportunity for us to help intervene in our patients' care. By being aware of triggers that result in worsening headaches, this provides an opportunity for the patient to affect their own outcome. And if information about triggers is presented to the patient early on, this knowledge will hopefully last them a lifetime.

## Data Availability

Not required.

## References

[1] Abu‐Arafeh I , Morozova M . Migraine in children and adolescents: Assessment and diagnosis. Handb Clin Neurol. 2024; 199: 475–85. 38307664 10.1016/B978-0-12-823357-3.00029-X

[2] Zahran E , Ahmed MAS . Patient and parent reported triggers of migraine attacks in children and adolescents. Dev Med Child Neurol. 2026; 68: 1476–1481. 10.1111/dmcn.70375 PMC13545497 42338281

